# Neonatal rebound hyperkalemia associated with ritodrine alone: a case report

**DOI:** 10.1186/s12887-021-02840-8

**Published:** 2021-08-31

**Authors:** Keita Osumi, Kenichi Suga, Masashi Suzue, Ryuji Nakagawa, Shoji Kagami

**Affiliations:** 1 Department of Pediatrics, Tokushima University Hospital, 2-50-1 Kuramotocho, Tokushima, 770-8503 Japan; 2 Department of Neonatology, Takatsuki General Hospital, 1-3-13 Kosobe-cho, Takatsuki, Osaka, Japan

**Keywords:** Neonatal hyperkalemia, Ritodrine, Betamimetic, Insulin, Tocolysis, Preterm

## Abstract

**Background:**

Betamimetics have been used for tocolysis extensively in the past, and one of them, ritodrine is widely used in Japan. Various adverse events have been reported for this agent, including newborn hypoglycemia and hypokalemia, as well as maternal hypokalemia and rebound hyperkalemia; however, cases of neonatal rebound hyperkalemia are not described in the literature.

**Case presentation:**

A male infant born at 36 weeks of gestation by cesarean section at a local maternity clinic suddenly entered cardiopulmonary arrest with ventricular tachycardia and fibrillation due to hyperkalemia (K^+^, 8.7 mmol/L). No monitoring, examination of blood electrolyte levels, or infusions had been performed prior to this event. Maternal infusion of ritodrine (maximum dose, 170 μg/min) had been performed for 7 weeks prior to cesarean section. After resuscitation combined with calcium gluconate, the infant died at 4 months old due to serious respiratory failure accompanied by acute lung injury following shock. No cause of hyperkalemia other than rebound hyperkalemia associated with ritodrine was identified.

**Conclusions:**

This case report serves as a warning regarding the potential risk of neonatal rebound hyperkalemia in association with maternal long-term ritodrine administration.

## Background

In Japan, the betamimetic ritodrine is often infused for longer than 48 h as tocolytic therapy. Long-term maternal administration of ritodrine is known to be associated with various adverse effects in newborns, including hypoglycemia, ileus, hypotension and hypocalcemia [1]. Previous reports have described rebound hyperkalemia in association with cessation of ritodrine in parturients [2], and Yada et al. recently reported that combined ritodrine and magnesium sulphate (MgSO_4_) raised the risk of neonatal hyperkalemia based on nationwide cohort research conducted in Japan [3]. However, neonatal rebound hyperkalemia associated with ritodrine alone has not been reported in the English literature.

This case report describes serious arrhythmia due to neonatal rebound hyperkalemia following maternal use of ritodrine alone.

## Case presentation

A Japanese male infant, weighing 2504 g, was born in a private hospital to a primiparous mother by emergency cesarean section at 36^+ 3/6^ weeks of gestation due to breech presentation. His mother had received continuous infusion of ritodrine for threatened preterm labor (maximum dose, 170 μg/min; tolerable limit, 200 μg/min) during the 7 weeks from 29^+ 3/6^ weeks of gestation to 1 h before cesarean section. The mother’s serum potassium level was 4.0 mEq/L at 1 day before cesarean section and 4.5 mEq/L at 5 days after cesarean section. Apgar score was 8 at 1 min and 10 at 5 min. After birth, the neonate was placed in an incubator without infusion or electrocardiogram (ECG) or saturation of percutaneous oxygen (SpO_2_) monitoring. The next day, mild grunting developed; SpO_2_ was 100% in room air, but electrolytes and blood gas analyses were not conducted. He was fed 10–20 mL of infant formula 5 times and urinated 4 times before the event.

**Fig. 1:**
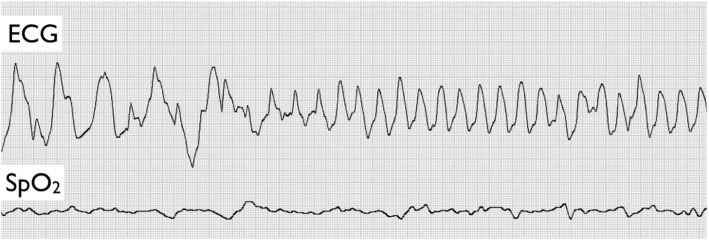
Electrocardiogram monitoring at admission, showing ventricular tachycardia to ventricular fibrillation. ECG, electrocardiogram; SpO_2_, percutaneous oxygen saturation

At 26 h old, sudden deoxygenation (SpO_2_, 77%) was detected, and oxygen administration was initiated*.* Then, about 1 h later, the infant entered cardiopulmonary arrest and was transferred under chest compressions and bag-mask ventilation to the neonatal intensive care unit of Tokushima University Hospital (Tokushima, Japan). He was gasping on admission and was immediately intubated to allow mechanical ventilation. Endobronchial bleeding was observed at suction. ECG monitoring showed ventricular tachycardia to ventricular fibrillation (Fig. 1). Ventricular tachycardia ceased temporarily with cardioversion at 6 J but soon recurred. Blood examination revealed hyperkalemia (K^+^, 8.7 mmol/L), hypoglycemia (glucose, 17 mg/dL), and severe metabolic acidosis (pH 6.94; base excess − 19.6 mmol/L). Administration of calcium gluconate successfully resolved the tachycardia. Neonatal rebound hyperkalemia was diagnosed, presumably associated with maternal ritodrine use.

**Fig. 2:**
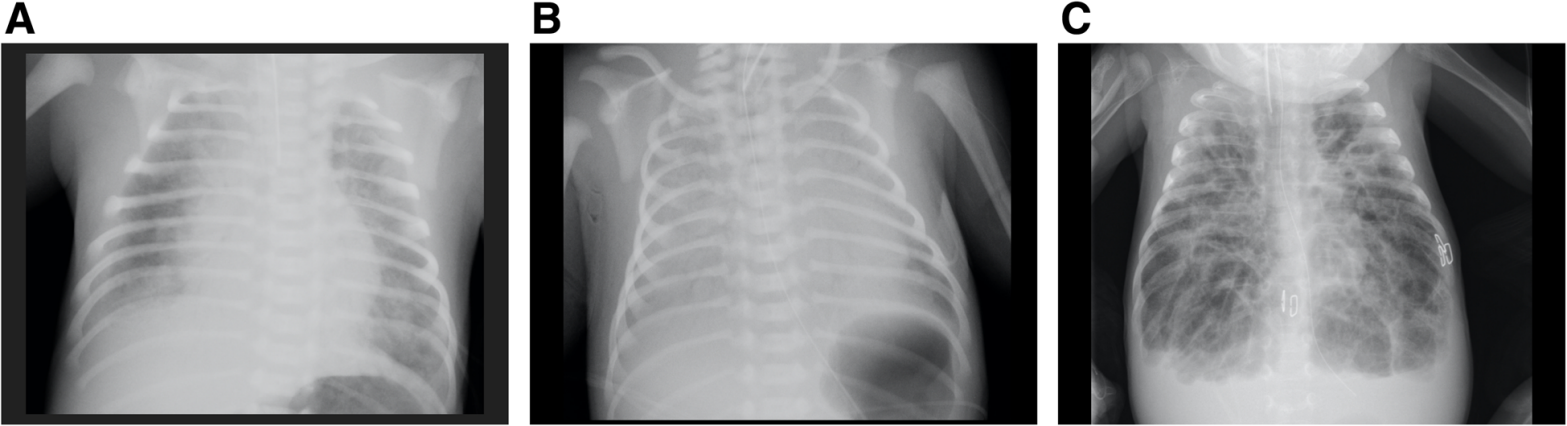
**A** Chest X-ray at admission. **B** Chest x-ray on the second day of hospitalization, showing progressive bilateral diffuse opacities. **C** Chest X-ray at 84 days of age

After resuscitation, hypoxemia under a fraction of inspired oxygen (FiO_2_) of 1.00 was observed (SpO_2_, lower limb showed 78–81%; upper limb 81–85%; oxygen index, 21–24.) Chest X-ray revealed bilateral diffuse irregular opacities (Fig. 2A, B). Cardiac ultrasound showed deteriorated left ventricular movement with 11.8% fractional shortening, massive tricuspid regurgitation (pressure gradients, 54 mmHg, as estimated from a systolic peak velocity of 3.7 m/sec), and bidirectional shunt (right-to-left dominant) in the ductus arteriosus, indicating persistent pulmonary hypertension. He was diagnosed as neonatal acute respiratory distress syndrome (nARDS) and required high-frequency oscillatory ventilation with inhaled nitric oxide at 30 ppm. Hyperkalemia, hypoglycemia, and metabolic acidosis were quickly normalized and did not recur. Newborn screening by tandem mass spectrometry revealed no abnormalities. Although steroid treatment was initiated, serious respiratory failure continued with dependence on mechanical ventilation at a high setting (FiO_2_, 0.6–0.9; mean airway pressure, 14–17 cmH_2_O; and delta pressure 60–90 cmH_2_O; SLE 5000 neonatal ventilator with high-frequency oscillator; SLE, Bridge Business Park, UK) with inhaled nitric oxide at 10 ppm. Chest X-ray showed serious overinflation (Fig. 2C). At 52 days old, left tension pneumothorax with cardiac arrest led to hypoxic ischemic encephalopathy after resuscitation and drainage of the thoracic cavity. The infant died due to severe respiratory failure at 97 days old. Autopsy was not performed because his parents declined consent.

## Discussion and conclusions

The present case report describes a late preterm infant who suddenly entered cardiac arrest due to arrhythmia. Certainly, hyperkalemia is seen after chest compression, but in the present case, neonatal hyperkalemia associated with maternal ritodrine usage resulting in arrhythmia and sudden cardiac arrest seems most likely for the following reasons. First, similar cases have been reported in the Japanese literature involved in twins born at full-term with symptomatic hyperkalemia to a mother who had received ritodrine for 7 weeks, until the day of delivery [4] (Table 1). These newborns presented with serum K^+^ of 9.8 mmol/L at 20 h old and K^+^ of 8.3 mmol/L at 19 h old, respectively, and both cases of hyperkalemia improved without recurrence with intravenous insulin plus glucose. Second, we did not identify any other potential causes of sudden cardiopulmonary arrest, such as congenital long QT syndrome, congenital Table 1.

**Table 1:** Data from the literature and present study regarding clinical presentation of newborns with symptomatic hyperkalemia associated with maternal ritodrine use

Author	GW	BW (g)	Onset (h)	K^+^ (mmol/L)	Symptoms	Outcome
Present case	36	2504	27	8.7	VT, VF	Death
Takayanagi^4)^	37	2866	20	9.8	Wide QRS, bradycardia	Survived healthy
	37	2814	19	8.3	Pallor, poor activity	Survived healthy

*GW* Gestational week, *BW* Body weight at birth, *VT* Ventricular tachycardia, *VF* Ventricular fibrillation

metabolic disorder, or pseudohypoaldosteronism [5]. Third, ventricular tachycardia and ventricular fibrillation in the patient we describe here were dramatically improved by administration of calcium gluconate, despite cardioversion proving ineffective, probably due to myocardial stabilization [6].

**Fig. 3:**
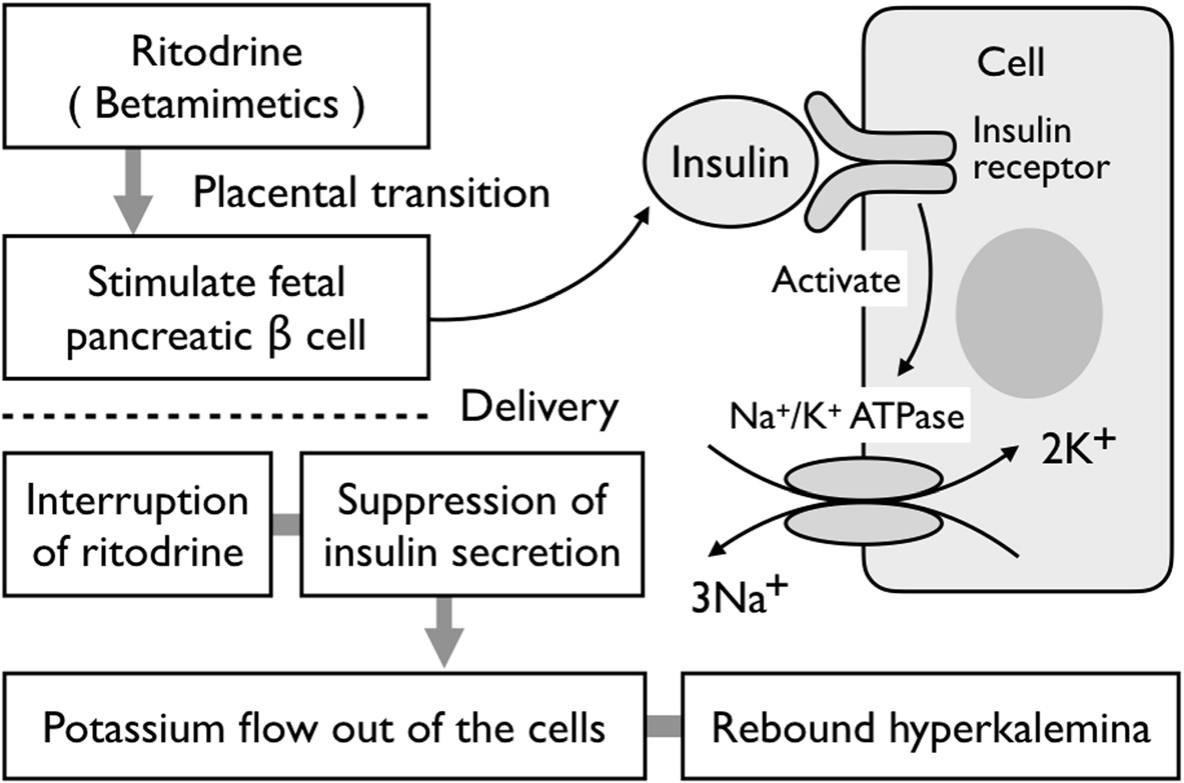
Mechanism of neonatal rebound hyperkalemia by maternal ritodrine administration. After passing through the placenta, ritodrine stimulates insulin secretion by the fetal pancreatic beta cells, which increases cellular uptake of extracellular potassium. When ritodrine is interrupted by parturition, it is thought that the resulting suppression of insulin secretion causes potassium efflux from cells

The postulated etiology of neonatal rebound hyperkalemia due to ritodrine is shown in Fig. 3. Ritodrine passes through the placenta and stimulates fetal pancreatic beta cells to facilitate the uptake of extracellular potassium into the cells by insulin. When the transplacental passage of ritodrine is interrupted at birth, insulin secretion is suppressed, and potassium starts to flow out of the cells [2]. This etiology was supported by a single-center retrospective study, in which serum potassium levels in a group of neonates whose mothers had been administered ritodrine were significantly higher than those in a non-tocolytic group at 12–24 h after birth [7]. Some reports have described puerperal rebound hyperkalemia associated with discontinuation of ritodrine via a similar mechanism [8–10].

In the present case, we diagnosed the observed acute lung injury following shock as nARDS in accordance with the Montreux definition proposed by De Luca et al. [11]: acute onset from a known or suspected clinical insult; exclusion of respiratory distress syndrome, transient tachypnea of newborns, and congenital anomalies; lung images with diffuse bilateral opacites or infiltrates; absence of congenital heart disease; oxygen deficit expressed as oxygen index. Cytokines associated with ischemia-reperfusion phenomena, oxidative stress, and ventilator-induced lung injury might exacerbate lung injury leading to death.

In the present case, the observed hypoglycemia on admission might have suppressed insulin secretion and exacerbate hyperkalemia, because glucose infusion alone improved the hyperkalemia. The hypoglycemia might have been a side effect of ritodrine or have been caused by no infusion despite the preterm status of the infant. The Causal Analysis Committee for Cerebral Palsy of the Japan Council for Quality Health Care (JCQHC) has identified several cases of cerebral palsy attributable to neonatal hyperkalemia and/or hypoglycemia, presumably associated with maternal tocolytic agents [12]. Following a request by the JCQHC, Yada et al. used nationwide data to investigate the relationships between neonatal hypoglycemia or hyperkalemia and maternal tocolytic agents [3]. The research showed that the occurrence of hypoglycemia was associated with the use of ritodrine with or without MgSO_4_, and that hyperkalemia was associated with concomitant use of ritodrine and MgSO_4_. No significant relationships were identified between neonatal hyperkalemia and mortality or neurological sequelae [3]. However, the sudden cardiac arrest and serious arrythmia in the present case suggest a potential risk of death from neonatal hyperkalemia associated with the use of ritodrine. According to systematic reviews [13], betamimetics are regarded as effective for delaying birth by up to 48 h, during which pregnant women can be transported to a higher-level hospital and can receive antenatal corticosteroids. However, no clinical trials have been conducted to determine whether long-term tocolytics improve fetal mortality and/or morbidity, so careful management is warranted with the long-term tocolysis conventionally performed in Japan [14, 15].

Late preterm infants are known to have elevated risks of apnea, hypoglycemia, transient tachypnea of the newborn, insufficient feeding, and neurological impairment [16]. As a result, tocolysis is often continued to near term, especially in local private hospitals outside neonatal intensive care units (NICUs), and no recommendations are made regarding the optimal gestational age in which to discontinue tocolysis in the clinical guidelines issued by the Japan Society of Obstetrics and Gynecology [15, 17]. Late preterm infants are usually cared for in step-down neonatal units or obstetric wards without close examination that is provided in NICUs. Considering that the half-life of ritodrine ranges from 4.2 h to 29.6 h, with ritodrine remaining at clinical relevant levels for up to 24–48 h after birth [18], monitoring of ECGs and serum K^+^ levels should be performed at least for this period, and episode of serum K^+^ exceeding 6.0 mEq/L should be noted [5].

The present case report is limited by the absence of data on serum potassium level before cardiac arrest and serum insulin level on admission. There is currently no direct evidence, in humans and animal models, of rebound hyperkalemia being caused by ritodrine or any other betamimetics. Furthermore, serious nARDS might have been tried extracorporeal membrane oxygenation for lung rest [19].

In conclusion, this case report provides a warning regarding the potential risk of neonatal rebound hyperkalemia in association with maternal long-term ritodrine administration.

### Recommendation

We recommend serum potassium measurement and ECG monitoring in newborns whose mothers have received long-term administration of ritodrine.

## Data Availability

Data that support the findings of this report are available from the corresponding author upon reasonable request.

## Abbreviations

ECG: Electrocardiogram
FiO_2_: Fraction of inspired oxygen;
JCQHC: Japan Council for Quality Health Care
MgSO_4_: Magnesium sulphate
nARDS: Acute respiratory distress syndrome
NICU: Neonatal intensive care unit
SpO_2_: Percutaneous oxygen saturation
